# Assessment of unilateral cleft lip and palate management using presurgical NAM

**DOI:** 10.6026/973206300221009

**Published:** 2026-02-28

**Authors:** Ashish Kalawat, Gaurav Jasoria, Sudhir Kumar, Saurabh Singh Parihar, Vaibhav Tammanna Chougule, Arpit Shrivastava, Rajat Misurya

**Affiliations:** 1 Department of Orthodontics and Dentofacial Orthopaedics, Maharaja Ganga Singh Dental College and Research Centre, Sri Ganganagar, Rajasthan, India; 2 Department of Plastic Surgery, Maharani Laxmi Bai Government Medical College, Jhansi, Uttar Pradesh, India; 3 Department of Orthodontics, Maharana Pratap College of Dentistry and Research Centre, Gwalior, Madhya Pradesh, India; 4 Department of Pediatric and Preventive Dentistry, Bharati Vidyapeeth (Deemed to be University) Dental College and Hospital, Sangli, Maharashtra, India; 5 Department of Dentistry, Maharani Laxmi Bai Medical College Jhansi, Uttar Pradesh, India

**Keywords:** Alveolar cleft width, cleft management, NAM therapy, nasoalveolar molding (NAM), unilateral cleft lip and palate

## Abstract

Unilateral cleft lip and palate is a complex craniofacial deformity involving disruption of the lip, alveolus, maxilla and nasal framework. Presurgical nasoalveolar molding (NAM) has emerged as an important adjunctive modality. Therefore, it is of interest to evaluate the effectiveness of presurgical nasoalveolar molding in infants with unilateral cleft lip and palate. 20 non-syndromic infants with unilateral cleft lip and palate underwent presurgical NAM therapy prior to primary lip repair. Presurgical nasoalveolar molding is an effective, non-invasive adjunct in the management of unilateral cleft lip and palate. Early initiation of NAM significantly optimizes presurgical anatomy.

## Background:

Cleft lip and palate constitute one of the most prevalent congenital craniofacial anomalies globally. Unilateral cleft lip and palate representing a complex deformity involving the lip, alveolus, maxilla and nasal framework. The burden of cleft anomalies is particularly significant in developing countries such as India. Delayed presentation, limited access to multidisciplinary cleft care and socioeconomic constraints often influence treatment outcomes. The severity of the initial cleft deformity plays a critical role. It helps in determining surgical complexity, esthetic results and the likelihood of secondary corrective procedures later in life [1, 2]. Primary surgical repair of unilateral cleft lip and palate performed without presurgical orthopedic intervention is frequently challenged. Wide alveolar clefts, nasal asymmetry, columellar deviation and increased tension across lip segments are influencing success. These anatomical discrepancies may adversely affect surgical precision and wound healing. This leads to compromised nasal esthetics, scar hypertrophy and a higher incidence of revision surgeries [3, 4]. Cleft management has highlighted the importance of presurgical interventions in reducing the severity of the deformity. Presurgical nasoalveolar molding (NAM) is a non-invasive orthopedic technique. The biological basis of NAM lies in the transient plasticity of neonatal cartilage attributed to elevated levels of maternal estrogen. This allows gradual and controlled molding of the alveolar segments and nasal cartilages [4, 5]. NAM facilitates tension-free surgical repair and enhances the potential for favorable esthetic and functional outcomes [6].

Numerous clinical studies have demonstrated the short-term benefits of NAM therapy in unilateral cleft lip and palate patients. It helps in reduction of alveolar cleft width, improved nasal symmetry, elongation of the columella and improved lip segment alignment [7, 8-9]. These presurgical improvements reduce the need for extensive surgical manipulation of the nasal cartilages [10]. Despite these advantages, the literature presents variability in treatment protocols, appliance design, outcome measures and duration of NAM therapy. This resulting in heterogeneity across reported outcomes [11]. Moreover, concerns regarding the long-term effects of NAM on maxillary growth have led to continued debate. Recent systematic reviews and meta-analyses, however, have provided considerable evidences. Which suggest that, presurgical NAM when appropriately administered and monitored, reinforcing its role as a safe adjunct in cleft management [1, 2]. In the Indian clinical setting, reports on presurgical NAM therapy largely consist of case reports and small cohort studies, with limited prospective observational studies [12, 13-14]. Clinical evidence is essential to substantiate the effectiveness of NAM therapy and guide clinical decision-making. Recent comprehensive clinical perspectives have highlighted the need for structured evaluation of NAM outcomes [15]. Therefore, it is of interest to assess the efficiency of presurgical nasoalveolar molding in the management of unilateral cleft lip and palate. Evaluation for changes in alveolar cleft width, nasal symmetry, columellar length and lip segment approximation prior to primary surgical repair was done.

## Materials and Methods:

The present prospective clinical observational study was designed with careful consideration of both statistical rigor and practical feasibility. Sample size estimation was performed with help of formula to calculate for a single population. Mean (n=Z^2^×SD^2^/d^2^) (n = Z^ {2} \times SD^ {2} / d^ {2}) (n=Z^2^×SD^2^/d^2^). Where n denotes the required sample size, Z represents the standard normal deviate corresponding to a 95% confidence interval (1.96). SD refers to the standard deviation derived from previously published NAM outcome studies. d indicates the allowable error. Based on prior Indian and international literature documenting a standard deviation ranging from 1.2 to 1.5 mm for alveolar cleft width reduction following NAM therapy and assuming a precision of 0.6 mm, the estimated sample size ranged between 16 and 18 subjects. To compensate for potential attrition and to strengthen the robustness and reliability of clinical assessment, the final sample size was rounded off to 20 infants. With Institutional Ethics Committee approval, the study was carried out in a tertiary care dental teaching hospital in India in the Department of Orthodontics and Dentofacial Orthopedics in cooperation with the Department of Oral and Maxillofacial Surgery. Prior to participation, all enrolled infants' parents or legal guardians provided written informed consent. A total of 20 infants diagnosed with non-syndromic unilateral cleft lip and palate, aged between 7 days and 3 months, were consecutively recruited based on predefined inclusion and exclusion criteria. Infants presenting with bilateral clefts, syndromic associations, systemic illnesses, or anticipated poor parental compliance were excluded from the study. All selected subjects undergo presurgical NAM therapy prior to primary lip repair as part of a comprehensive cleft management protocol.

Primary impressions were obtained using elastomeric addition silicone impression material under stringent pediatric monitoring to ensure safety, following which a customized NAM appliance was fabricated using heat-polymerized acrylic resin. The appliance comprised an intraoral molding plate aimed at gradual approximation of the alveolar segments, along with an integrated nasal stent assembly designed to facilitate progressive nasal cartilage remodeling. Appliance retention was achieved using extra-oral adhesive tapes applied across the cleft lip segments and periodic adjustments were performed at weekly intervals to incrementally guide alveolar alignment and nasal form correction. Clinical parameters including alveolar cleft width, nasal symmetry, columellar length and lip segment approximation were recorded at baseline and after completion of NAM therapy, immediately prior to surgical intervention. Standardized clinical photographs and dental casts were utilized for documentation and all measurements were carried out using digital calipers to ensure precision, consistency and reproducibility. The collected data were subjected to statistical analysis by utilizing SPSS software, with p<0.05 as the suitable for statistical significance.

## Results:

All 20 infants enrolled in the study successfully completed presurgical Nasoalveolar Molding therapy and no appliance-related complications or dropouts were observed during the study period. Quantitative assessment of clinical parameters demonstrated a consistent and statistically significant improvement following NAM therapy. At baseline, the mean alveolar cleft width was relatively wide, reflecting the severity of unilateral cleft presentation. Following completion of NAM therapy, a marked reduction in alveolar cleft width was observed, indicating effective approximation of the alveolar segments. Similarly, nasal symmetry scores and columellar length exhibited a significant increase post-therapy, reflecting favorable nasal cartilage molding and midline correction. Lip segment approximation also showed notable improvement prior to primary surgical repair (Table 1). Overall, presurgical NAM therapy resulted in statistically significant improvements across all evaluated parameters. Paired statistical comparison of pre- and post-treatment values was carried out using the paired t-test. The analysis revealed a highly significant reduction in alveolar cleft width following NAM therapy (p< 0.001). Additionally, improvements in nasal symmetry, columellar length and lip segment approximation were found to be statistically significant, further confirming the effectiveness of presurgical NAM in improving both alveolar and nasal morphology prior to surgical intervention (Table 2).

## Discussion:

The present prospective clinical observational research evaluated the effectiveness of presurgical nasoalveolar molding (NAM) in the management of infants with unilateral cleft lip and palate. This study assessed changes in alveolar cleft width, nasal symmetry, columellar length and lip segment approximation prior to primary surgical repair. The findings demonstrated statistically significant improvements across all evaluated parameters following NAM therapy, highlighting its efficacy as an adjunctive presurgical intervention. A pronounced and statistically significant reduction in alveolar cleft width was observed following NAM therapy, indicating effective approximation of the alveolar segments. This finding is consistent with previous studies and systematic reviews that have reported substantial narrowing of the alveolar cleft as one of the primary benefits of NAM, thereby facilitating tension-free surgical repair and improving surgical precision [1, 2-3]. Reduction in alveolar gap prior to surgery has been shown to simplify operative techniques and may contribute to improved postoperative healing and esthetic outcomes [4, 5].

In addition to alveolar changes, the present study demonstrated considerable enhancement in nasal symmetry and columellar length following NAM therapy. These findings support the biological rationale of NAM, which exploits the increased plasticity of neonatal nasal cartilage during early infancy to achieve gradual nasal reshaping [4, 6]. Improved nasal symmetry and columellar elongation observed in this study are in agreement with previous clinical investigations reporting favorable nasal form and reduced nasal deformity severity prior to cheiloplasty [7, 8-9]. Such improvements may reduce the extent of surgical manipulation required during primary lip repair and potentially lower the need for secondary nasal corrective procedures. The statistically significant enhancement in lip segment approximation observed in this study further underscores the role of NAM in optimizing presurgical anatomy. Improved alignment of lip segments contributes to reduced surgical tension at the time of repair, which has been associated with improved scar quality and lip esthetics [10-11]. Collectively, these presurgical improvements reflect the comprehensive orthopedic and molding effects of NAM on both hard and soft tissue components of the cleft deformity. Despite the demonstrated short-term benefits, the long-term effects of NAM on maxillary growth remain a subject of debate. While earlier studies raised concerns regarding potential growth restriction, recent systematic reviews and meta- analyses have reported no significant adverse effects on midfacial growth when NAM is appropriately administered and monitored [1, 2]. These findings support the continued use of NAM as a safe presurgical modality within a multidisciplinary cleft care framework. In the Indian clinical context, where access to specialized cleft care may be limited and delayed presentation is common, the role of NAM assumes particular importance.

Previous Indian studies have largely been confined to case reports and small series, reporting favorable outcomes but lacking standardized quantitative evaluation [3, 12, 13-14]. The present study adds to the existing Indian literature by providing objective, statistically validated clinical evidence supporting the effectiveness of presurgical NAM therapy in unilateral cleft lip and palate infants. Nevertheless, the limitations of this study must be acknowledged. The comparatively smaller sample size and lack of a non-NAM control group restrict the generalizability of the findings. Additionally, the study focused on short-term presurgical outcomes and did not assess long-term facial growth or postoperative surgical results. Future researches with larger sample sizes, comparative designs and long-term follow-up are warranted to further substantiate the clinical benefits of NAM and refine treatment protocols [8].

## Clinical and managerial implications:

From a clinical perspective, the results of this study reinforce presurgical nasoalveolar molding as an effective, non-invasive adjunct in the comprehensive management of unilateral cleft lip and palate. NAM therapy significantly improves alveolar alignment, nasal symmetry, columellar length and lip segment positioning prior to surgery, thereby simplifying primary surgical repair and potentially enhancing esthetic and functional outcomes. Early initiation of NAM within the neonatal period should be emphasized as part of standardized cleft treatment protocols in tertiary care centers [4, 7]. From a managerial and healthcare delivery standpoint, incorporation of NAM therapy into routine cleft care pathways may contribute to reduced surgical complexity, shorter operative times and potentially decreased need for secondary corrective surgeries, thereby optimizing resource utilization. Establishing trained multidisciplinary cleft teams, improving parental counseling and compliance and integrating NAM services into government and institutional cleft care programs could enhance overall treatment efficiency and patient outcomes, particularly in resource-constrained settings such as India [8, 11]. Yadav *et al.* stated that NAM helps in approximation of the alveolar segments before surgical procedure [15].

## Advancement to knowledge with NAM management:

By actively molding the alveolus, lip and nose before to surgery using neonatal cartilage plasticity, presurgical Nasoalveolar Molding (NAM) improves therapy for unilateral cleft lip and palate (UCLP). NAM reduces the need for secondary revision procedures by improving symmetry and especially addressing the nasal deformity (*e.g.*, by raising the drooping alar cartilage). NAM reduces a large, severe cleft to a tiny one, providing an excellent basis for surgical reconstruction.

## Conclusion:

Presurgical nasoalveolar molding is an effective adjunct in the management of unilateral cleft lip and palate, producing significant improvement in alveolar approximation, nasal symmetry, columellar length and lip segment alignment. Early initiation of NAM optimizes presurgical anatomy, facilitates primary surgical repair and may enhance overall esthetic and functional outcomes. Thus, we show the routine incorporation of NAM into comprehensive cleft care protocols, particularly in the Indian clinical setting.

## Figures and Tables

**Table 1:** Clinical parameters before and after NAM therapy

**Parameter**	**Baseline (Mean ± SD)**	**Post-NAM (Mean ± SD)**
Alveolar cleft width (mm)	8.42 ± 1.36	3.21 ± 0.98
Nasal symmetry score	1.82 ± 0.41	3.14 ± 0.52
Columellar length (mm)	4.26 ± 0.73	6.18 ± 0.81
Lip segment approximation (mm)	2.14 ± 0.62	4.87 ± 0.74

**Table 2:** Comparison of clinical parameters before and after NAM therapy

**Parameter**	**Mean Difference**	**t-value**	**p-value**
Alveolar cleft width (mm)	5.21	12.84	<0.001*
Nasal symmetry score	1.32	8.46	<0.001*
Columellar length (mm)	1.92	9.18	<0.001*
Lip segment approximation (mm)	2.73	10.02	<0.001*
Statistically significant at p< 0.05
